# Supplementary material for: Prevalence and sociodemographic correlates of anogenital Human Papillomavirus (HPV) carriage in a cross-sectional, multi-ethnic, community-based Asian male population
Source: PLoS One. 2021 Jan 20;16(1):e0245731. doi: 10.1371/journal.pone.0245731 (PMC7817061; doi:10.1371/journal.pone.0245731)
Supplement: S1 Table — (DOCX) [file pone.0245731.s001.docx]

S1 Table: Comparing sample sufficiency between clinician-collected samples and participant self-collected samples

| Sampling Method | Genital Sample Sufficiency, n (%) | | | Anal Sample Sufficiency, n (%) | | |
| --- | --- | --- | --- | --- | --- | --- |
|  | No | Yes | p-value | No | Yes | p-value |
| Clinician Sampling | 58 (21.0) | 218 (79.0) | **0.039** | 86 (31.2) | 190 (68.8) | **<0.001** |
| Self-sampling | 27 (13.6) | 171 (86.4) |  | 19 (9.6) | 179 (90.4) |  |

P-value <0.05 is considered statistically significant and is marked in bold font.

P-value is generated using Pearson Chi Square Test.
