# Supplementary material for: Prevalence and sociodemographic correlates of anogenital Human Papillomavirus (HPV) carriage in a cross-sectional, multi-ethnic, community-based Asian male population
Source: PLoS One. 2021 Jan 20;16(1):e0245731. doi: 10.1371/journal.pone.0245731 (PMC7817061; doi:10.1371/journal.pone.0245731)
Supplement: S2 Table — (DOCX) [file pone.0245731.s002.docx]

**S2 Table: Comparing sample sufficiency across different types of sampling device**

| Sampling device | Genital Sample Sufficiency, n (%) | | | Anal Sample Sufficiency, n (%) | | |
| --- | --- | --- | --- | --- | --- | --- |
|  | No | Yes | p-value | No | Yes | p-value |
| Hybribio Cytobrush | 44 (18.1) | 199 (81.9) | 0.959 | 58 (23.9) | 185 (76.1) | **<0.001** |
| ‘Just For Me’ Cervical Sampler | 23 (17.2) | 111 (82.8) |  | 42 (31.3) | 92 (68.7) |  |
| Qiagen DNA Pap Cervical Sampler | 18 (18.6) | 79 (81.4) |  | 5 (5.2) | 92 (94.8) |  |

P-value <0.05 is considered statistically significant and is marked in bold font.

P-value is generated using Pearson Chi Square Test.
